# Comparative Analysis of Differentially Expressed Genes and Metabolites in Waxy Maize Inbred Lines with Distinct Twin-Shoot Phenotypes

**DOI:** 10.3390/plants14131951

**Published:** 2025-06-25

**Authors:** Mengfan Qin, Guangyu Li, Kun Li, Jing Gao, Meng Li, Hao Liu, Yifeng Wang, Keke Kang, Da Zhang, Wu Li

**Affiliations:** 1 Guangdong Province Key Laboratory of Crop Genetic Improvement, Crop Research Institute, Guangdong Academy of Agricultural Sciences, Guangzhou 510640, China; 2 Changli Institute of Pomology, Hebei Academy of Agriculture and Forestry Sciences, Qinhuangdao 066600, China

**Keywords:** waxy maize, twin-shoot seedling, plant hormone, transcriptome, developmental network

## Abstract

Polyembryonic maize, capable of producing multiple seedlings from a single kernel, holds great potential value in agricultural and industrial applications, but the seedling quality needs to be improved. In this study, seedlings of two waxy maize (*Zea mays* L. *sinensis* Kulesh) inbred lines, D35 (a polyembryonic line with twin shoots) and N6110 (single-shoot), exhibited similar relative growth rates during 1 to 5 days post-germination. UPLC-MS/MS profiling of 3- to 5-day-old seedling roots and shoots revealed that H2JA, MeSAG, and IAA-Val-Me were the common differentially accumulated metabolites (DAMs) of the 3-day-old vs. 5-day-old seedlings of D35 and N6110 in the same tissues, and MeSAG, tZ9G, cZROG, and DHZROG were identified in D35 vs. N6110 across the same tissues and the same periods. RNA-seq analyses showed various processes involved in seedling development, including DNA replication initiation, rhythmic processes, the cell cycle, secondary metabolic processes, and hormone biosynthetic regulation. The differentially expressed genes (DEGs) between D35 and N6110 were significantly enriched in organic hydroxy compound biosynthetic, alcohol biosynthetic, organic hydroxy compound metabolic, abscisic acid biosynthetic, and apocarotenoid biosynthetic processes. The KEGG-enriched pathways of DAMs and DEGs identified that *AUX1*, *AHP*, *A-ARR*, *JAR1*, *SIMKK*, *ERF1*, and *GID2* might be conserved genes regulating seedling growth. The integrated analyses revealed that 98 TFs were potentially associated with multiple hormones, and 24 of them were identified to be core genes, including 11 AP2/ERFs, 4 Dofs, 2 bZIPs, 2 MADS-box genes, 2 MYBs, 1 GATA, 1 LOB, and 1 RWP-RK member. This study promotes a valuable understanding of the complex hormone interactions governing twin-shoot seedling growth and offers potential targets for improving crop establishment via seedling quality.

## 1. Introduction

Polyembryonic maize has emerged as a noteworthy maize type in recent years due to its capacity to produce multiple individuals from a single kernel, endowing it with significant agronomic and industrial value [1]. Compared to non-polyembryonic maize, polyembryonic kernels exhibit higher levels of tryptophan and lysine, an improved protein quality, enhanced ratios of fatty acids (e.g., oleic and linoleic acids), elevated potassium and phosphorus contents [2,3,4,5], and better physicochemical, phytochemical, techno-functional, and antioxidant properties [4,5,6]. Polyembryonic maize sprouts have higher hydrolysable polyphenol content, condensed tannin content, and better nutritional and nutraceutical properties [6,7]. However, seedlings from polyembryonic seeds generally exhibit lower vigor and weaker soil-breaking abilities, which affects seedling emergence in the field. Furthermore, suboptimal growth during the seedling stage can compromise the entire growth cycle, leading to diminished yields or weakened stress tolerance. Therefore, it is essential to investigate the intrinsic regulatory factors involved in the growth of polyembryonic maize seedlings.

Plant hormones are signaling molecules that orchestrate various facets of plant growth, development, and environmental adaptation, influencing processes such as seed germination and maturation, vegetative and reproductive growth, and embryonic development. The principal hormones that have been studied in depth include auxin (IAA), cytokinin (CK), gibberellin (GA), abscisic acid (ABA), ethylene (ETH), brassinosteroid (BR), jasmonate (JA), salicylic acid (SA), and strigolactone (SL). Each hormone exerts distinct influences on plant growth, yet there is considerable interplay and overlap in the biological processes [8,9,10,11,12,13,14,15,16]. IAA, CK, GA, BR, and SL play essential roles in plant development, while ABA, ET, JA, and SA are known to mediate plant biotic and abiotic stresses [17,18]. Auxin is the earliest discovered member of the phytohormone family, critically regulating root stem cell maintenance primarily through inducing the accumulation of transcription factors *PLT1/2* [19,20]. Cytokinin type-B response factors directly activated *WUSCHEL* expression, thereby modulating stem cell activity [21,22,23]. Overexpression of the type-A response regulator *OsRR2* enhanced root growth, whereas RNAi of *OsRR2* significantly suppressed root development in rice (*Oryza sativa* L.) [24]. Lovastatin treatment (cytokinin biosynthesis inhibitor) markedly inhibited adventitious root growth, demonstrating the positive regulatory role of cytokinin on root architecture [25]. JA inhibited primary root elongation via *MYC2*-mediated transcriptional repression of *PLT1/2* through direct promoter binding [26] and orchestrated lateral root formation by modulating auxin biosynthesis and polar transport [27]. There are similarities between different hormone signaling pathways. For example, the signaling pathways of IAA, JA, SL, and CK demonstrated similarities in that they were subject to feedback regulation through the recruitment of co-repressors *TOPLESS (TPL)/TOPLESS-RELATED (TPR)* by ethylene-responsive element binding factor-associated Amphiphilic Repression (EAR) motif-containing transcriptional repressors such as *AUX/IAA*, *JAZ-NINJA*, and *SMXL/D53*, which enables accurate control of hormonal actions [28,29,30,31]. Therefore, fine crosstalk between different hormones is regulated around the entire reproductive cycle of the plant.

Overall, recent years have witnessed remarkable advances in the understanding of hormonal signaling, but endogenous hormone levels and associated networks specific to polyembryonic maize seedlings remain unclear. This study employed morphological characterization, hormone profiling, and transcriptomic analyses on two waxy maize lines (*Zea mays* L. *sinensis* Kulesh). D35, a polyembryonic maize line, could produce two equally sized shoots from a single seed, and N6110 was a normal single-shoot inbred line. Through these integrated analyses, this research aimed to elucidate the molecular mechanisms governing shoot development and to predict the hormonal-gene networks operating in twin-shoot seedlings. This study could provide valuable insights for improving the quality of twin-shoot seedlings and exploring the regulatory network of hormone signaling during the seedling stage.

## 2. Results

### 2.1. Morphology of N6110 and D35 Seedlings

**Figure 1:**
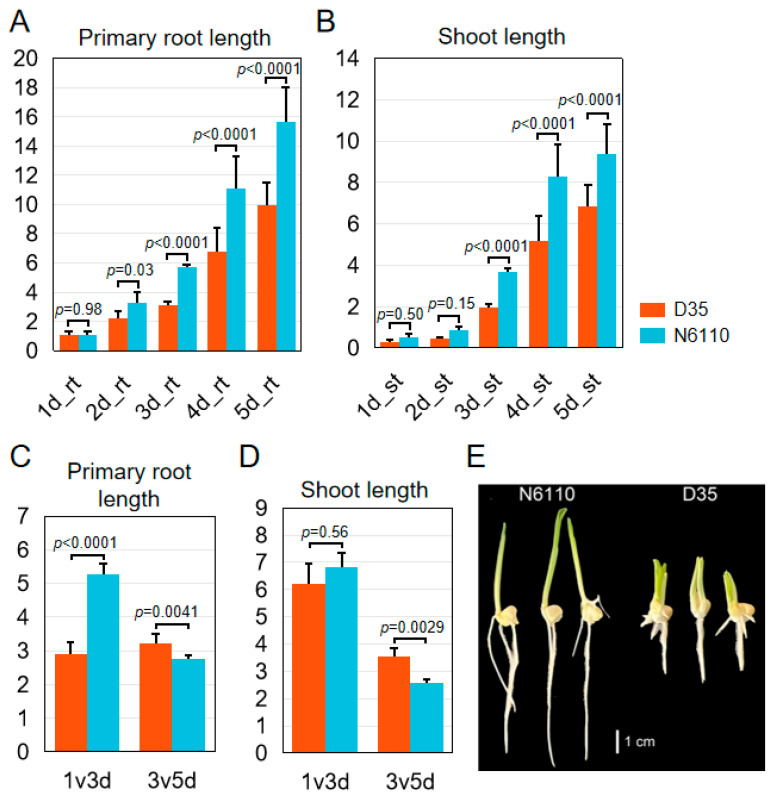
Morphological statistical analyses on the 1st-5th days after germination for N6110 and D35. Three plants of each inbred line are displayed. (**A**) primary root length and (**B**) shoot length of N6110 and D35 seedlings from the 1st day to the 5th day post-germination. Relative change rate of (**C**) primary root length and (**D**) shoot length. The data are the means ± standard deviations (SDs) of five plants. For D35, only one of the twin shoots was counted. Two-way ANOVA calculated the *p*-value with Tukey’s HSD test. (**E**) Photos of N6110 and D35 on the 3rd day after germination.

The morphologies of N6110 and D35 seedlings exhibited significant morphology differences under normal conditions, with D35 developing two shoots and primary roots (Figure 1). In root development, while no significant differences were detected in the primary root length between D35 and N6110 on day one (1.06 cm vs. 1.08 cm, *p* = 0.98), gradual divergence became apparent, with D35 ultimately exhibiting shorter primary roots compared with N6110 (*p* < 0.05) (Figure 1A). In regard to shoot development, D35 and N6110 exhibited significant differences in the initial shoot length on day one (0.31 cm vs. 0.53 cm), which might be attributed to the initial length of embryo germs (Figure 1B). While D35 and N6110 did not exhibit significant phenotypic alterations in consecutive two-day intervals (Supplementary Figure S1), their divergence demonstrated statistically significant differentiation when evaluated across the third to fifth day after germination (Figure 1C,D). Given that D35 develops two functional shoots compared to N6110 (Figure 1E), the consistency of seedling growth regulation between it and the single shoot line has not been reported in previous studies. We would like to explore the intrinsic hormonal and transcriptional networks underlying these phenotypic divergences during early seedling growth.

### 2.2. Analysis of Different Hormone-Related Compounds

**Figure 2:**
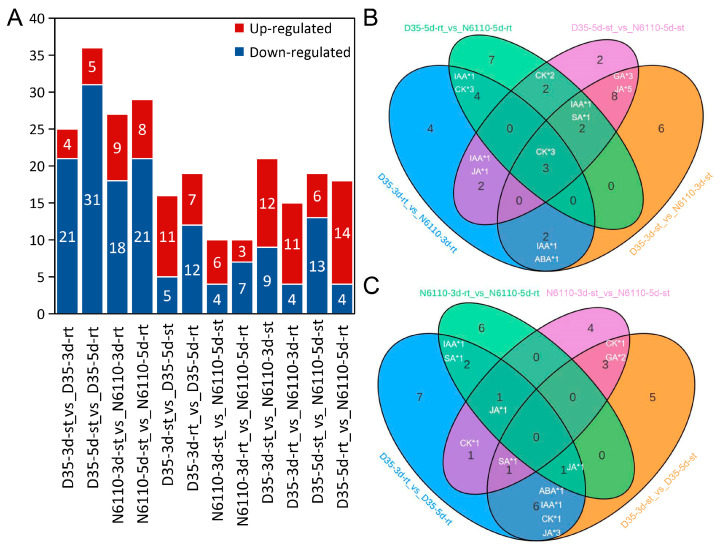
Profiles of the hormone compounds in N6110 and D35. (**A**) The number of DAMs identified in different tissues and at different seedling stages. Red and blue represent the up- and down-regulated DAMs, respectively. The selection criteria were determined by the variable importance in projection (VIP) values, FDR (false discovery rate), and fold changes. (**B**) Venn diagram of DAMs shared between N6110 and D35 among the same tissues in the same stages. (**C**) Venn diagram of DAMs shared between 3- and 5-day-old seedlings in roots and shoots of the same lines. The number and type of DAMs are displayed in the figures. rt: root; st: shoot.

To detect the different hormones or active compounds that are responsible for the growth of N6110 and D35 seedlings, we conducted phytohormone assays on roots and shoots on the third and fifth days after germination. A total of 109 hormone-related compounds were assayed in the 24 samples, including auxins, CKs, ABAs, JAs, SA, GAs, SLs, ETH, and MLT (Supplementary Table S1), but only 55 of them had detectable abundances. Over 80% of the substances exhibited a Coefficient of Variation (CV) of less than 0.2 in quality-control samples, and a high degree of correlation was observed among biological replicates (Supplementary Figure S2). In total, 50 differentially accumulated metabolites (DAMs) were identified across various comparative combinations, including 18 CKs, 13 auxins, 7 GAs, 9 JAs, 2 SAs, and 1 ABA (Figure 2 and Supplementary Table S2). Among these classes of hormones (Supplementary Figure S4), the compounds from the same tissue at the same time were more abundant in D35 than in N6110, and the contents of ETHs, ABA, CKs, and GAs showed significant differences between the two lines, indicating their significant roles in seedling development.

**Figure 3:**
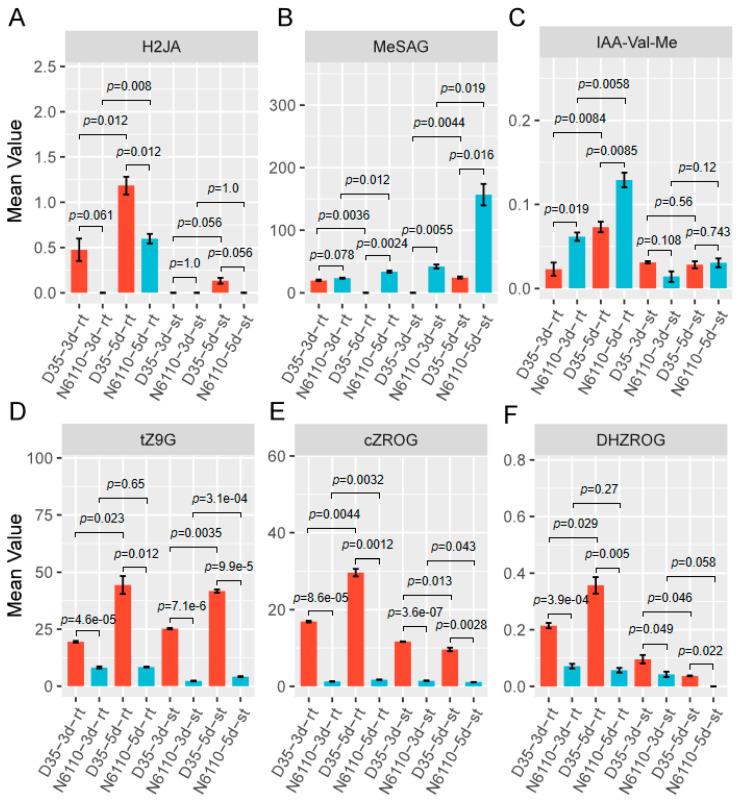
Contents of the six DAMs in the 24 samples. (**A**–**F**) were the contents of H2JA, MeSAG, IAA-Val-Me, MeSAG, tZ9G, cZROG, and DHZROG, respectively. The data are shown as the means ± standard errors (SEs) with three replicates, and the unit on the Y-axis is ng/mg. H2JA: Dihydrojasmonic acid; MeSAG; 2-Methoxycarbonylphenyl beta-D-glucopyranoside; IAA-Val-Me: Indole-3-acetyl-L-valine methyl ester; tZ9G: trans-Zeatin-9-glucoside; cZROG: cis-Zeatin-O-glucoside riboside; DHZROG: Dihydrozeatin-O-glucoside riboside.

Among these DAMs, 24 compounds (ABA, cZR, DHZ7G, DHZROG, GA20, GA53, H2JA, IAA, IAA-Asp, IAA-Glc, IAA-Glu, IAA-Val-Me, ILA, iP9G, IPR, MEIAA, MEJA, MeSAG, OPC-4, OPDA, SAG, TRA, and tZRMP) were identified as DAMs in at least three comparative analyses between roots and stems of D35 and N6110 (Supplementary Figure S4). Meanwhile, three DAMs (H2JA, MeSAG, and IAA-Val-Me) were identified as DAMs from 3 to 5 days with at least three comparative analyses, and four DAMs (MeSAG, tZ9G, cZROG, and DHZROG) showed varying accumulation patterns between N6110 and D35 in the same tissues and periods, with at least three comparative analyses (Figure 3).

Among these DAMs, 24 compounds (ABA, cZR, DHZ7G, DHZROG, GA20, GA53, H2JA, IAA, IAA-Asp, IAA-Glc, IAA-Glu, IAA-Val-Me, ILA, iP9G, IPR, MeIAA, MeJA, MeSAG, OPC-4, OPDA, SAG, TRA, and tZRMP) were identified as DAMs in at least three comparative analyses between roots and stems of D35 and N6110 (Supplementary Figure S4). Meanwhile, three DAMs (H2JA, MeSAG, and IAA-Val-Me) were determined as DAMs from 3 to 5 days with at least three comparative analyses. Four DAMs (MeSAG, tZ9G, cZROG, and DHZROG) exhibited distinct accumulation patterns between N6110 and D35 when comparing the same tissues and developmental stages, with these differences being consistently observed in at least three comparative analyses (Figure 3). Notably, concentrations of H2JA, tZ9G, cZROG, and DHZROG were significantly elevated in D35 compared to corresponding tissues of N6110. Conversely, IAA-Val-Me was lower in D35 roots than in N6110, and no differences in shoots.

### 2.3. Transcriptome Analysis for Seedlings

To study genome-wide gene expression in different tissues of N6110 and D35 at the third and the fifth days post-germination, we performed RNA-seq of the above plant samples. Approximately 1,217,487,590 raw reads were generated from 24 samples, and the average Q30 (an error rate of 0.1%) was 94.92% (Supplementary Table S3). After filtering and trimming the raw reads, 1,153,826,040 high-quality reads were used for further analysis, and the average genome mapping rate was 89.69%. A total of 42,877 expressed genes were identified, and among those, 9058 were novel genes. The Pearson correlation analysis and principal component analysis (PCA) results showed that the three biological replicates in the two inbred lines were closely clustered, indicating that the variation within the replicates at each time point is acceptable (Supplementary Figure S5).

**Figure 4:**
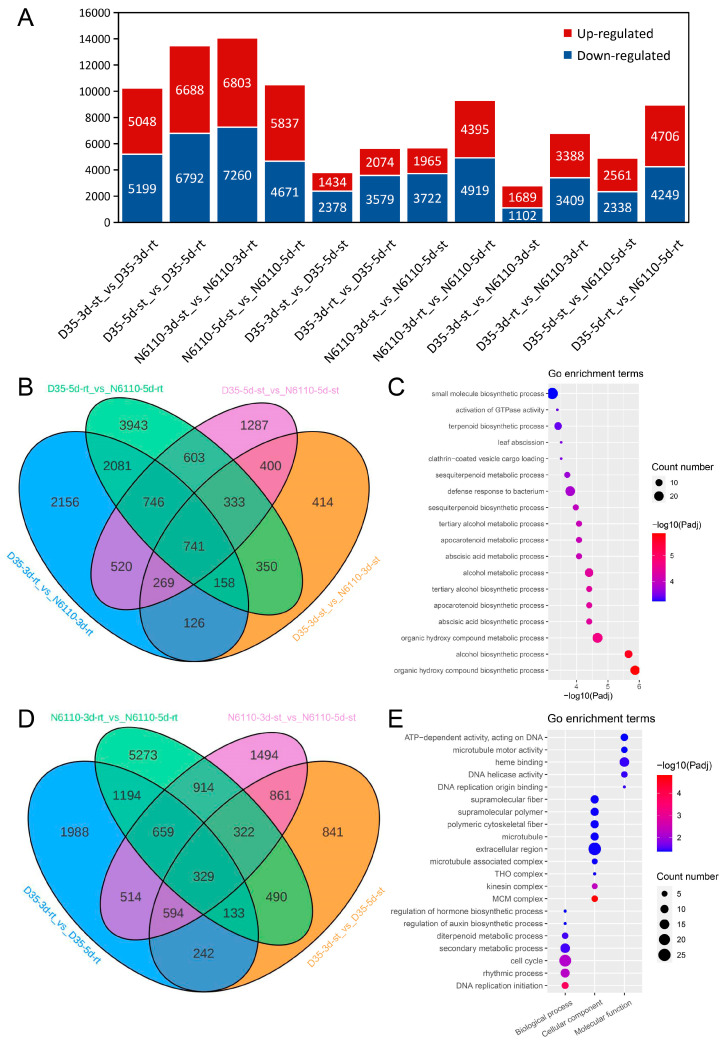
Genome-wide gene expression changes in 24 RNA-seq samples. (**A**) Number of differentially expressed genes (DEGs) identified in different tissues during different seedling stages. The selection criteria of *FDR* < 0.05 and |log_2_(FoldChange)| ≥ 1 were used. (**B**) Venn diagram of DEGs shared between N6110 and D35 among the same tissues in the same stages. (**C**) GO enrichments for the 741 DEGs. Only biological process terms are displayed. (**D**) Venn diagram of DEGs shared between 3- and 5-day-old seedlings in roots and shoots of the same lines. (**E**) GO enrichments for 329 DEGs. In this panel, red and blue indicate small and big *padj* (*padj* < 0.05), respectively. The bigger the bubble, the more annotated genes. rt: root; st: shoot.

The identified DEGs showed a significant change in gene expression, with a more than two-fold change (false discovery rate, FDR < 0.05). A total of 10,247, 13,480, 14,063, and 10,508 DEGs were identified from comparisons between the root and shoot (D35-3d-st_vs_D35-3d-rt, D35-5d-st_vs_D35-5d-rt, N6110-5d-st_vs_N6110-3d-rt, and N6110-5d-st_vs_N6110-5d-rt, respectively) (Supplementary Figure S6). Between days 3 and 5, 3812, 5653, 5687, and 9314 DEGs were identified in the following comparisons: D35-3d-st_vs_D35-5d-st, D35-3d-rt_vs_5d-rt, N6110-3d-st_vs_N6110-5d-st, and N6110-3d-rt_vs_N6110-5d-rt, respectively (Figure 4A), and a total of 2791, 6797, 4899, and 8955 DEGs were identified, respectively, in the following comparisons: D35-3d-st_vs_N6110-3d-st, D35-3d-rt_vs_N6110-3d-rt, D35-5d-st_vs_N6110-5d-st, and D35-5d-rt_vs_N6110-5d-rt (Figure 4A).

For the same tissues during the same period, 3726 and 1743 DEGs were identified between N6110 and D35 in roots and shoots, respectively. A total of 741 DEGs were shared in roots and shoots; these may be genes that regulate seedling growth (Figure 4B). These genes were mainly enriched in organic hydroxy compound biosynthetic processes (GO:1901617), alcohol biosynthetic processes (GO:0046165), organic hydroxy compound metabolic processes (GO:1901615), abscisic acid biosynthetic processes (GO:0009688), and apocarotenoid biosynthetic processes (GO:0043289) (Figure 4C and Supplementary Table S4). KEGG enrichment analysis for them showed that they were significantly enriched in glutathione metabolism (ko00480), carotenoid biosynthesis (ko00906), glucosinolate biosynthesis (ko00966), metabolic pathways (ko01100), and tyrosine metabolism (ko00350), among others (Supplementary Table S5).

For the same tissues of the same lines, 2315 and 2106 DEGs between 3 and 5 days after germination were identified in roots and shoots, respectively, and N6110 and D35 shared 329 DEGs 3 days after germination compared to those observed 5 days after germination (Figure 4D). These genes were significantly enriched in DNA replication initiation (GO:0006270), the rhythmic process (GO:0048511), the cell cycle (GO:0007049), secondary metabolic processes (GO:0019748), and the regulation of hormone biosynthetic processes (GO:0046885) (Figure 4E and Supplementary Table S4). Furthermore, these genes were significantly enriched in KEGG pathways involving sesquiterpenoid and triterpenoid biosynthesis (ko00909), DNA replication (ko03030), phenylpropanoid biosynthesis (ko00940), and the circadian rhythm (ko04712) (Supplementary Table S5); these pathways may influence growth differences between the two lines.

### 2.4. KEGG Pathway Co-Enrichments of DAMs and DEGs

**Figure 5:**
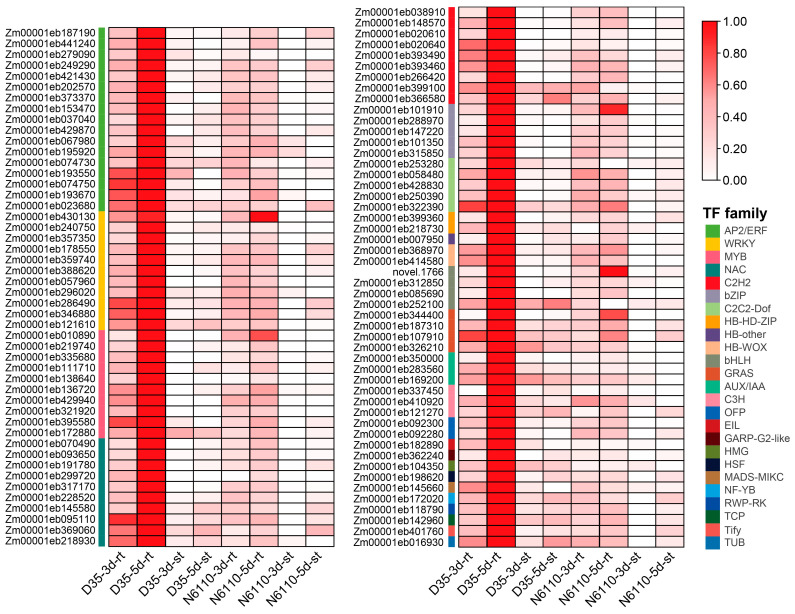
Heatmap of 98 differentially expressed TFs identified in the correlation network. The FPKM values were normalized from zero to one. Different TF families are displayed in various colors.

To explore relevant networks of seedling growth compounds and genes in both N6110 and D35, correlation analyses were performed using quantitative values of the genes and metabolites in all samples. Results with correlation coefficients greater than 0.8 in absolute value and with a *p*-value less than 0.05 were selected. In this study, we were mainly concerned with differences across species and between periods; thus, six DAMs (IAA-Val-Me, H2JA, MeSAG, tZ9G, cZROG, and DHZROG) were further analyzed in this study. These DAMs were also correlated with each other (|*r*| ranged from 0.02 to 0.92) (Supplementary Figure S7). A total of 3213 DEGs with high correlation coefficients to the six DAMs were screened out. These genes were mainly enriched in phenylpropanoid biosynthesis (ko00940, *padj* = 1.87 × 10^−6^), plant–pathogen interactions (ko04626, *padj* = 3.43 × 10^−6^), plant hormone signal transduction (ko04075, *padj* = 1.32 × 10^−5^), photosynthesis (ko00195, *padj* = 5.66 × 10^−5^), biosynthesis of secondary metabolites (ko01110, *padj* = 3.13 × 10^−4^), and glutathione metabolism (ko00480, *padj* = 5.75 × 10^−3^) (Supplementary Figure S8A). A total of 317 TFs were identified in the correlation network, and 98 of them were significantly associated with various DAMs (Supplementary Figure S8). Most of them were members of the AP2/ERF, WRKY, NAC, C2H2, and MYB families (Figure 5), indicating that these families might play a role in multi-hormone interactions. Most gene expressions were significantly positively correlated with DAM profiles, with higher expressions in roots than shoots, higher expressions in D35 than in N6110, and lower expressions after 3 d than after 5 d (Supplementary Figure S8B).

**Figure 6:**
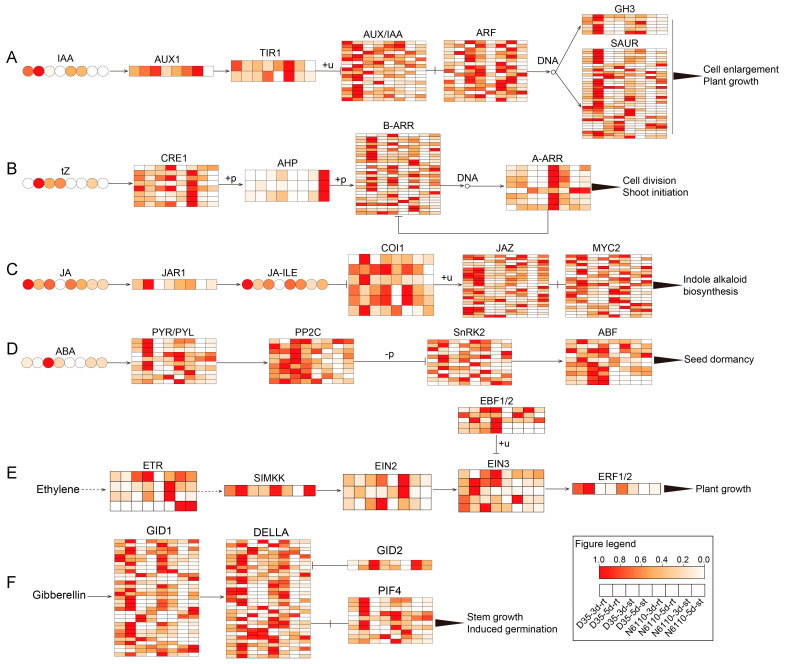
The DAMs and DEGs involved plant hormone signal transduction pathways. (**A**–**F**) Auxin, cytokinin, jasmonic acid, abscisic acid, ethylene, and gibberellin transduction pathways. Hormone abundance and gene expression are represented by heat maps, with darker colors indicating higher expression. The values were normalized from zero to one. Circles represent hormone compounds, and genes are represented by boxes. rt: root; st: shoot.

We further analyzed the significantly enriched KEGG pathways for DAMs with DEGs whose plant hormone signal transduction was closely related to plant hormone metabolism, and drew the pathways (Figure 6). A total of five compounds (IAA, ABA, tZ, JA, and JA-ILE) and 428 genes (217 of them TFs) were involved in these hormone pathways (Supplementary Table S6). Among these genes, we found *AUX1* (*Zm00001eb026490*), *AHP* (*Zm00001eb153570*, *Zm00001eb283590*, and *Zm00001eb353520*), *A-ARR* (*Zm00001eb066570*, *Zm00001eb182130*, *Zm00001eb405030*, *Zm00001eb433500*, *Zm00001eb082130*, and *Zm00001eb140730*), *JAR1* (*Zm00001eb358430*), *SIMKK* (*novel.5828*), *ERF1* (*Zm00001eb307550*), and *GID2* (*Zm00001eb401080* and *Zm00001eb008130*) showed the same trends in expression between the third day and the fifth day but different trends between N6110 and D35. In addition, *A-ARR* (*Zm00001eb247920*) showed the same trends in the two lines. These genes might be conserved genes that regulate seedling growth and development.

### 2.5. Prediction of Hormone-Gene Relation Networks

**Figure 7:**
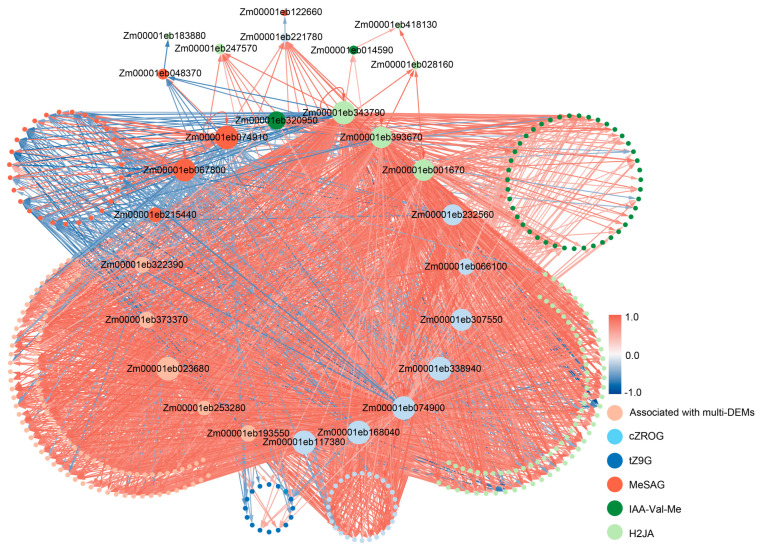
Core network construction for DEGs based on binding site prediction and correlation analyses. Edges with arrows represent direction. Different colored nodes represent that the genes were correlated with different DAMs, and brownish-pink nodes are correlated with multiple DAMs. The color of the edge represents the correlation coefficient between the two nodes, with blue being negative and red being positive.

In pursuit of a deeper understanding of the shared TFs across various plant hormone signaling pathways, we analyzed the binding sites of 317 TFs on the gene promoter sequences of 3213 DEGs. A total of 24 TFs were predicted to bind to the promoter regions of other TFs, while 283 TFs showed no evidence of binding sites (Figure 7). These 24 genes included 11 AP2/ERFs, 4 Dofs, 2 MADS-box genes, 2 MYBs, 2 bZIPs, 1 GATA, 1 LOB, and 1 RWP-RK (gene information is presented in Table 1), suggesting that they may act as crucial regulatory hubs within the network. Furthermore, it is worth noting that of the 47 TFs in the plant hormone signal transduction pathway, most did not have detectable binding sites, except for *Zm00001eb307550*. This indicated that these hormone-specific TFs might be regulated by other TFs, which highlights the complexity of hormone signaling pathways.

### 2.6. RNA-Seq Expression Validation via qRT-PCR

**Figure 8:**
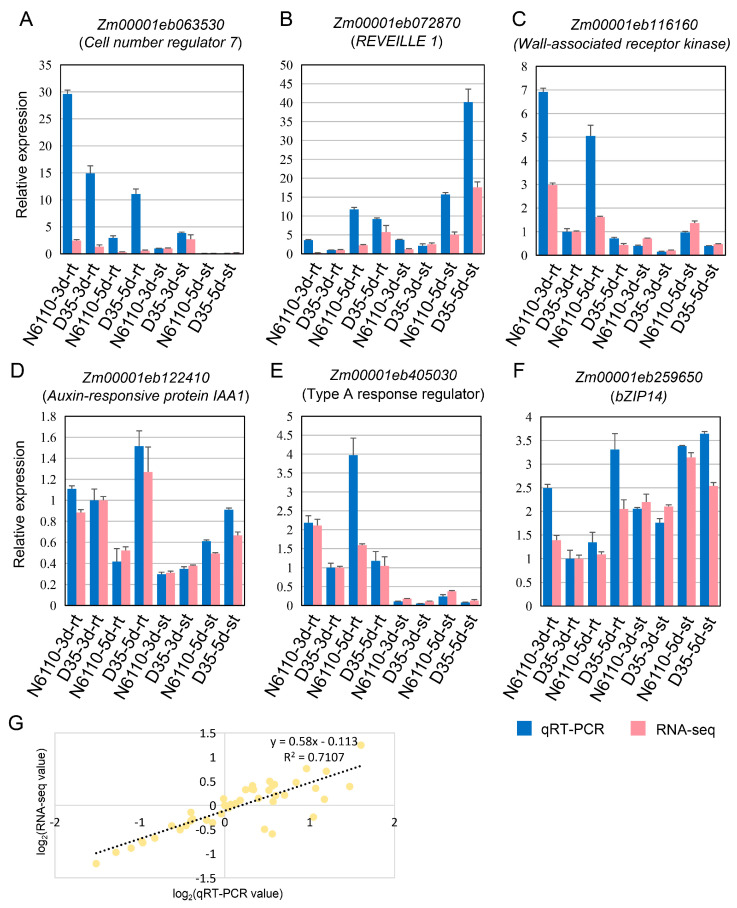
Validation of RNA-seq data reliability using quantitative real-time PCR. (**A**–**F**) Relative expressions of the six genes by qRT-PCR and RNA-seq. Relative expression is calculated as the value of D35-3d-rt as a standard, and the data are shown as the means ± SDs. The x-axis represents the samples, and the y-axis shows the fold change in relative gene expression. rt: root; st: shoot. (**G**) Correlation analysis of expression values of RNA-seq and qRT-PCR. The scatter plot shows the relationship between the gene expression values measured by RNA-seq and qRT-PCR. The X-axis represents the log_2_(qRT-PCR value) of the qRT-PCR, and the Y-axis represents the log_2_(RNA-seq value) of the RNA-seq. The fitted line equation indicates a strong linear relationship between these two methods.

To verify the gene expression reliability of RNA-seq, the gene expressions of six DEGs were detected by qRT-PCR, including three genes from the plant hormone signal transduction pathways, one circadian rhythm gene, one wall-associated gene, and one cell number regulator (Supplementary Table S7). The results showed that the expression trends of these genes via qRT-PCR were consistent with those via RNA-seq data (Figure 8), confirming the reliability of the RNA-seq analysis results in this study.

## 3. Discussion

Hormonal assays demonstrated that among the significantly altered hormone classes in the same tissues between D35 (a polyembryonic maize inbred line exhibiting twin shoots) and N6110 (normal control) on the third and fifth days after germination, the differential abundances of hormones in roots were predominantly CKs and IAAs, whereas those in shoots encompassed CKs, IAAs, GAs, JAs, and SAs. In this study, D35 exhibited significantly higher cytokinin content concurrent with shorter root and shoot lengths and reduced relative growth rates compared to N6110 (Figure 1). This observation aligns with previous studies suggesting that excessive cytokinin accumulation can suppress root and stem elongation [25,32,33,34], potentially contributing to the growth differences observed between these specific genotypes. Only IAA-Val-Me exhibited differential accumulation across inbred lines and developmental stages among the 13 auxin-active compounds detected (Supplementary Table S2). Notably, N6110 roots contained significantly higher IAA-Val-Me levels than D35, whereas D35 demonstrated greater primary root relative elongation rates from days 3–5. IAA-Val-Me was a kind of storage form of IAA [35], and IAA-Val was biologically active like free IAA, inducing plant developmental responses in root and hypocotyl elongation inhibition [36]. Previous studies have shown higher levels of free IAA in senescent leaves than in non-senescent leaves [37,38], implying that IAA-Val-Me may have a negative effect on root growth in waxy maize.

The JA and SA pathways play crucial roles in plant growth. Previous studies have shown that the *jaz* decuple mutant (*jazD*; *jaz1/2/3/4/5/6/7/9/10/13*) exhibits severe growth inhibition [39], whereas inhibition of JA biosynthesis enhances cell elongation capacity [40]. In this study, the significantly higher levels of H2JA content in D35 were observed alongside its specific growth characteristics. H2JA was a type of jasmonate and might promote callus formation and multiplication [41,42,43]. Thus, it might be relevant to the role of root and shoot elongation in D35 and warrants further investigation. In this study, two salicylic acid (SA)-active compounds were detected, SAG and MeSAG (Supplementary Table S2). MeSAG, identified as a non-volatile storage form (MeSA 2-O-β-d-glucoside) of MeSA [44], showed significantly higher levels in N6110 compared to D35 (Figure 3). Previous studies indicate that SA overaccumulation can induce growth dwarfism [45,46], and MeSAG has been implicated as a potential growth-inhibitory DAM in other studies [47,48,49]. This suggested a possible growth inhibition of MeSAG levels in waxy maize seedlings.

Plant hormone signal transduction has its own specific signaling pathways and core genes. In this study, plant hormone signal transduction (ko04075) pathways were enriched by both DAMs and DEGs. In these pathways, we identified several genes that have been validated in maize, such as *AUX1*, *A-ARR*, and *GID2*. Many studies have shown that the apical meristems are the site of auxin synthesis; the auxin is then transported to other parts of the plant through transporter proteins such as AUX and PIN and co-regulates plant growth and development with other hormones [8,50]. *A-ARRs* are negative regulators of cytokinin signaling, and disruption of eight of the Arabidopsis A-ARRs altered the levels of PIN proteins and increased sensitivity to N-1-Naphthylphthalamic acid [51,52]. GA3 application could reduce the expression of *GID2*, an F-box subunit of the SCF E3 complex [53]. *GID2* could regulate the gibberellin-dependent degradation of SLR1 [54] and interacts with P7-2, which functions in gibberellin, auxin, jasmonate, ethylene, and light response processes [55]. Here, the differential expression patterns of *ZmAUX1* (*Zm00001eb026490*), *ZmA-ARR* (*Zm00001eb066570*, *Zm00001eb182130*, *Zm00001eb405030*, *Zm00001eb433500*, *Zm00001eb082130*, and *Zm00001eb140730*), and *ZmGID2* (*Zm00001eb401080* and *Zm00001eb008130*) between D35 and N6110, despite similar trends across days within each line, highlighted genotype-specific regulation of these core hormone signaling components. Their known functions in auxin transport, cytokinin signaling modulation, and gibberellin response make them candidates potentially contributing to the observed physiological differences between these inbred lines.

Cross-regulation between hormone signaling pathways is essential for plant growth and development, and the sharing of transcription factors is one of the major modes of hormone cross-regulation [56]. In this study, we constructed a regulatory network of DEGs associated with IAA-Val-Me, TRA, DHZROG, tZ9G, cZROG, and H2JA, and identified 24 core TFs in the network, which included 11 *AP2/ERF* members, 4 *Dof* members, 2 *bZIP* members, 2 MADS-box members, 2 *MYB* members, 1 *GATA* member, 1 *LOB* member, and 1 *RWP-RK* member. All of these transcription factor families have been strongly associated with hormonal signaling pathways in previous studies [57,58,59,60,61,62,63], suggesting that these 24 TFs may be the key genes that participate in the hormonal signaling transduction pathways. *Zm00001eb307550* encoded an ethylene-responsive factor 1 (*ERF1*), the only TF predicted to have binding sites in the promoter regions of other TFs. This phenomenon may arise from the fact that a large number of the regulatory target genes of TF were far from detectable TF binding sites [64]. In the previous studies, *ERF1* served as an upstream component in both ET and JA signaling, with its expression being rapidly induced by ET or JA stimuli [65]. ERF1 could promote auxin transport by up-regulating *PIN1* and *AUX1* expression while inhibiting *ARF7* transcription, thereby integrating environmental signals to facilitate local auxin accumulation with a modified distribution [66]. The ABI3-ERF1 regulatory loop was implicated in lateral root emergence, in which the physical interaction between ABI3 and ERF1 can modulate the expression of genes regulated by ERF1-ABI3, such as *PIN1*, *AUX1*, *ARF7*, and *ABI5* [67]. ERF1 serves as a bridge linking the ET signaling pathway to the JA, IAA, and ABA pathways. Although *ERF1* was up-regulated in the coleoptile of Zheng58 seedlings under blue light [68], its function was still unclear in maize. In this study, the expression of *Zm00001eb307550* was highly correlated with DHZROG content (Table 1), implying that *ERF1* might also play a role in the cytokinin pathway.

In summary, this comparative analysis revealed significant genotype-specific differences in hormone metabolites and gene expression profiles between the twin-shoot waxy maize D35 and normal waxy maize N6110, which exhibit distinct twin-seedling phenotypes. These differences encompassed key hormone classes, signaling genes, and a co-expression network of DEGs associated with specific metabolites. Although these findings provided a molecular characterization that distinguished these two inbred lines during the seedling period and suggested potential associations with known hormone pathways and growth phenotypic differences, future functional validation using genetic and molecular biological approaches will be required. The candidate genes and metabolites identified in this study lay a crucial foundation for subsequent in-depth investigation into the molecular mechanisms underlying the development of polyembryonic maize seedlings.

## 4. Methods and Materials

### 4.1. Plant Material and Growth

Two waxy maize inbred lines, ‘N6110’ and ‘D35’, were obtained from the Crop Research Institute (CRI) of the Guangdong Academy of Agricultural Sciences (GDAAS). D35 was a polyembryonic line with two shoots of the same size from one kernel, and N6110 was a normal waxy maize with a single shoot. Seeds were surface sterilized using 2% NaClO for 30 min and then washed five times in pure water for germination using the paper-roll method. Morphological indices were measured from the 1st to the 5th days post-germination. The temperature of the incubator was maintained at 24 °C day/22 °C night, with a 16 h/8 h light/dark cycle. The root and shoot lengths of the plant individuals were measured in five plants with three replicates. The roots and shoots were harvested and frozen in −80 °C refrigerators for hormone detection and RNA sequencing. The differences between groups were determined by two-way ANOVA with Tukey’s HSD test by R v4.2.3. Data were displayed as means ± SEs of biological samples.

### 4.2. Determination of Endogenous Phytohormones

The methods of sample preparation and extraction were referred to in the previous studies [69]. Liquid nitrogen-ground sample powder (50 mg) was weighed into a pre-chilled 2 mL centrifuge tube. A 10 μL aliquot of internal standard mixture (100 ng/mL in methanol) and 1 mL of extraction solvent (methanol/water/formic acid, 15:4:1 *v*/*v*/*v*) were added sequentially. The mixture was vortexed vigorously for 10 min at 4 °C, followed by centrifugation at 12,000× *g* for 5 min (4 °C). The supernatant was transferred to a fresh tube through a 0.22 μm nylon filter and diluted 1:10 (*v*/*v*) with 80% (*v*/*v*) aqueous methanol. Processed samples were stored at −80 °C until LC-MS/MS analysis.

Standard solutions of 109 hormonal compounds were prepared in a gradient concentration series spanning 0.01–500 ng/mL. To enhance detection sensitivity for specific analytes, 5DS_1, H2JA, IAA-Glc+Na, SAG, ST, TRP, and t-CA_1 were formulated at 20× higher concentrations (0.2–10,000 ng/mL), while Phe solutions were prepared at 30× elevated levels (0.3–15,000 ng/mL). Quantitative analysis was performed using a triple quadrupole mass spectrometer operated in multiple reaction monitoring (MRM) modes of the LC-MS/MS platform, with isotopically labeled internal standards incorporated for signal normalization. Calibration curves were established by plotting the concentration ratio (external standard: internal standard) against the corresponding peak area ratio. All analytes exhibited excellent linear responses within their respective quantification ranges (R^2^ > 0.99, see Supplementary Table S1). Experimental samples were quantified by interpolating measured peak area ratios into these regression equations.

### 4.3. LC-MS/MS Platform and Settings

The LC-MS/MS platform for metabolite profiling consisted of an ultra-performance liquid chromatography system (ExionLC™ AD, Sciex, Marlborough, MA, USA) coupled with a hybrid triple quadrupole-linear ion trap mass spectrometer (QTRAP^®^ 6500+, Sciex, Marlborough, MA, USA). Chromatographic separation was achieved using a Waters ACQUITY UPLC HSS T3 C18 column (1.8 µm particle size, 100 mm × 2.1 mm i.d.) maintained at 40 °C. The mobile phase comprised (A) ultrapure water with 0.04% acetic acid and (B) acetonitrile with 0.04% acetic acid, delivered at a flow rate of 0.35 mL/min with the following gradient program: 95% A (0–1 min), linear gradient to 5% A (1–8 min), hold at 5% A (8–9 min), return to initial conditions (9.1 min), and column equilibration (9.1–12 min). The sample injection volume was 2 μL.

Mass spectrometric detection was performed using electrospray ionization (ESI) in both positive and negative modes with the following parameters: ion source temperature 550 °C, ion spray voltage 5500 V (positive mode)/−4500 V (negative mode), and curtain gas flow 35 psi. Compound-specific detection was achieved through multiple reaction monitoring (MRM) with optimized declustering potentials and collision energies for each ion transition pair.

### 4.4. Differential Analysis of Phytohormone Abundance

Z-score normalization was applied to the dataset, followed by hierarchical clustering analysis (HCA) and principal component analysis (PCA) across all samples. Based on the variable importance in projection (VIP > 1) values obtained from orthogonal partial least squares discriminant analysis, metabolites showing differences between groups were preliminarily screened, followed by further selection of *p* < 0.05 and |foldchange| ≥ 2. The annotated metabolites were retrieved from the KEGG compound database (http://www.kegg.jp/kegg/compound, accessed on 29 January 2024).

### 4.5. RNA Isolation and RNA-Seq Analyses

RNA was isolated using CTAB-PBIOZOL, quantified with a Qubit instrument, and further analyzed using a Qsep400 bioanalyzer. cDNA libraries were prepared and sequenced by the MetWare company (Wuhan, China, http://www.metware.cn/, accessed on 4 September 2024). Raw sequencing data underwent quality control with fastp v0.23.2 to remove adapters and low-quality reads [70]. Clean reads were aligned to the B73 NAM V5 reference genome using Hisat2 v2.2.1 with default settings [71]. Novel genes were identified using Stringtie v2.1.6, and gene expression levels were analyzed with FeatureCounts v2.0.3. FPKMs (fragments per kilobase of exon per million mapped fragments) were calculated for downstream analysis. The DEGseq2 v1.22.1 R package and selection criteria of *FDR* < 0.05 and |log_2_(FoldChange)| ≥ 1 were used to identify DEGs. GO term and KEGG pathway enrichment analyses were performed by ClusterProfile v4.6.0 [72]. Known *Arabidopsis* TF information was obtained from PlantTFDB, and DEG protein sequences were blasted to the PlantTFDB [73].

### 4.6. Network Construction of Hormone-Related DEGs

Different metabolites and genes were integrated into the KEGG pathway map for the shared KEGG pathways. Pearson correlations between DEGs and DAMs were analyzed across all samples, with |*r*| ≥ 0.8 and *p*-value < 0.05 used to identify significant associations for initial core network screening. Conserved motifs were identified based on TF protein sequences, and Fimo (https://meme-suite.org/meme/doc/fimo.html?man_type=web, accessed on 29 December 2024) was employed to analyze binding sites by linking these motifs to the 2000 bp promoter regions upstream of genes (*q*-value < 0.01 [74]). The core TF binding motifs to the gene promoter were listed in Table S8. The resulting networks were visualized using Cytoscape v3.10.1 [75].

### 4.7. Quantitative Real-Time PCR (qRT-PCR) Analysis

The RNA samples were reverse-transcribed into cDNA using a HiScript 1st strand cDNA synthesis Kit (Vazyme, Nanjing, China). The housekeeping gene GAPDH served as the internal reference control for normalizing expression data [76]. Primer pairs were designed via Primer-BLAST on NCBI and listed in Supplementary Table S9 and were used for qRT-PCR analysis. The qRT-PCR experiment was conducted using Taq Pro SYBR qPCR Master mix (Vazyme, Nanjing, China) on a Bio-Rad CFX96 system (Hercules, CA, USA) under the following conditions: initial denaturation at 95 °C for 30 s, followed by 40 cycles of denaturation at 95 °C for 3 s and annealing/extension at 60 °C for 10 s. Relative expression levels were calculated using the 2^−∆∆Ct^ method. Three biological and technical triplicates were performed to ensure data reliability and consistency.

## 5. Conclusions

In this study, we investigated two waxy maize inbred lines, D35 (polyembryonic maize having two shoots) and N6110 (single shoot), finding that the seedlings of D35 showed similar relative growth rates to N6110 during 1 to 5 days post-germination. Using UPLC-MS/MS analysis, we found that H2JA, MeSAG, and IAA-Val-Me were the DAMs from 3- to 5-day-old seedlings in the same tissues of D35 and N6110, and MeSAG, tZ9G, cZROG, and DHZROG were DAMs of D35 vs. N6110 in the same tissues of the same periods. Furthermore, RNA-seq analyses showed that DEGs between 3- and 5-day-old seedlings were involved in DNA replication initiation, rhythmic processes, the cell cycle, secondary metabolic processes, and hormone biosynthetic processes. DEGs between D35 and N6110 seedlings were enriched in the organic hydroxy compound biosynthetic process, alcohol biosynthetic process, organic hydroxy compound metabolic process, abscisic acid biosynthetic process, and apocarotenoid biosynthetic process. *AUX1*, *AHP*, *A-ARR*, *JAR1*, *SIMKK*, *ERF1*, and *GID2* were identified as possibly conserved genes regulating seedling growth and development via KEGG-enriched pathways of DAMs and DEGs. A total of 24 TFs were identified through TF binding site analyses that might be vital hub genes associated with hormonal networks. The identified key genes and metabolites in this study are potential targets for genetic improvement and crop management strategies to enhance seedling vigor and yield potential of polyembryonic maize.

## Figures and Tables

**Table 1:** Summary of 24 core TFs in gene expression–hormone networks of waxy maize.

Gene ID	Chr	Related DAMs	TF Family	Gene Annotation
*Zm00001eb023680*	chr1	DHZROG, cZROG	AP2/ERF	Ethylene-responsive transcription factor 5
*Zm00001eb067800*	chr2	MeSAG	AP2/ERF	EREBP-like factor
*Zm00001eb074900*	chr2	DHZROG	AP2/ERF	Ethylene-responsive transcription factor 6
*Zm00001eb074910*	chr2	MeSAG	AP2/ERF	EREBP-like factor
*Zm00001eb168040*	chr4	DHZROG	AP2/ERF	Ethylene-responsive transcription factor TINY
*Zm00001eb193550*	chr4	DHZROG, cZROG	AP2/ERF	Ethylene-responsive transcription factor RAP2-1
*Zm00001eb232560*	chr5	DHZROG	AP2/ERF	EREBP-like factor
*Zm00001eb307550*	chr7	DHZROG	AP2/ERF	Ethylene-responsive transcription factor 1
*Zm00001eb338940*	chr8	DHZROG	AP2/ERF	Ethylene-responsive transcription factor 4-like
*Zm00001eb343790*	chr8	H2JA	AP2/ERF	EREBP-like factor
*Zm00001eb373370*	chr9	H2JA, DHZROG	AP2/ERF	EREBP-like factor
*Zm00001eb014590*	chr1	IAA-Val-Me	bZIP	bZIP transcription factor 1/2/11/44/53
*Zm00001eb028160*	chr1	H2JA	bZIP	bZIP transcription factor 1/2/11/44/53
*Zm00001eb048370*	chr1	MeSAG	C2C2-Dof	--
*Zm00001eb066100*	chr2	DHZROG	C2C2-Dof	--
*Zm00001eb253280*	chr5	DHZROG, cZROG	C2C2-Dof	--
*Zm00001eb322390*	chr7	H2JA, DHZROG	C2C2-Dof	--
*Zm00001eb215440*	chr5	MeSAG	C2C2-GATA	FAR1 transcription factor
*Zm00001eb117380*	chr2	DHZROG	LOB	LOB domain-containing protein 16
*Zm00001eb001670*	chr1	H2JA	MADS-MIKC	MADS-box transcription factor
*Zm00001eb393670*	chr9	H2JA	MADS-MIKC	MADS-box transcription factor
*Zm00001eb221780*	chr5	DHZROG	MYB	Transcription factor MYB30
*Zm00001eb247570*	chr5	H2JA	MYB	Myb-related protein MYB4
*Zm00001eb320950*	chr7	IAA-Val-Me	RWP-RK	--

## Data Availability

The RNA-seq raw data generated in this study are available in SRA (https://www.ncbi.nlm.nih.gov/sra/ accessed on 29 January 2024) of NCBI with the accession number PRJNA1207110.

## Supplementary Materials

The following supporting information can be downloaded at https://www.mdpi.com/article/10.3390/plants14131951/s1, Figure S1: Relative growth rate from the 1st–5th day after germination; Figure S2: Quality control analyses conducted on 109 hormone compounds. Figure S3: Contents of different hormone classes of 50 DEMs in N6110 and D35; Figure S4: Venn diagram of DAMs between root and shoot shared in the same varieties; Figure S5: Statistical analysis of 24 samples based on genome-wide gene expression; Figure S6: Venn diagram of DEGs between root and shoot shared in the same varieties; Figure S7: Heatmap of Pearson correlation analyses for DEMs; Figure S8: Statistical analysis of DAMs and DEGs in correlation network; Figure S9: Network construction for DEMs and DEGs based on correlation analyses; Table S1: 109 hormone compounds information and calibration curve equation; Table S2: Differentially accumulated metabolites information and content; Table S3: Summary of RNA-seq data quality and alignment to reference in 24 samples; Table S4: GO enrichment analyses for DEGs; Table S5: KEGG enrichment analyses for DEGs; Table S6: Gene information for the DEGs identified in lant hormone signal transduction (ko04075) pathways; Table S7: Gene information for the qRT-PCR genes; Table S8: The TF binding motif of tht 24 TFs; Table S9: Primer pairs information for the qRT-PCR genes.
